# On the Structure of the Muscular Substance of the Heart

**Published:** 1850-12

**Authors:** 


*On the Structure of the Muscular Substance of the Heart.*—The following important observation is by Remak. The facts observed by him are best seen in the thin muscular layers which can be procured, especially in sheep, from the commencement of the great veins of the neck or the pulmonary veins. Two sets of muscular fibres can be distinguished; some which run parallel, and others which are between these and interlace, connecting the adjoining parallel fibres to each other. The net-work formed by the connecting or intermediate fibres differs in complexity in the different parts of the heart, auricles, ventricles, &c. Sometimes, instead of these intermediate connecting fibres, the sides of two parallel-running fibres approach, and fuse into each other (*eine partielle Verschmelzung der Rander zweier Hauptfasern.*) The intermediate fibres are often much smaller than the parallel, and of variable strength; occasionally, as in the ventricles, they are as large as the parallel fibres; it is then very difficult to make out the arrangement; yet in no place, either in the auricle or ventricle, is this arrangement wanting. This observation of Remak's has been confirmed in Wurzburg by Virchow.—*Ibid from Ibid.*

